# Supplementary material for: Identification of photoperiod-induced specific miRNAs in the adrenal glands of Sunite sheep (Ovis aries)
Source: Front Vet Sci. 2022 Jul 22;9:888207. doi: 10.3389/fvets.2022.888207 (PMC9354845; doi:10.3389/fvets.2022.888207)
Supplement: Supplementary file 1 [file Data_Sheet_1.ZIP › Supplementary file/miRDeep2 file.zip!/miRDeep2 file/Know_Structure/SP42A1/oar-mir-26b.pdf]

miRBase precursor : oar-mir-26b
Total read count : 127810
oar-miR-26b read count : 127703
remaining reads : 107

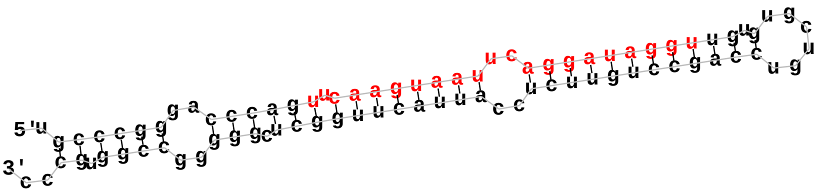

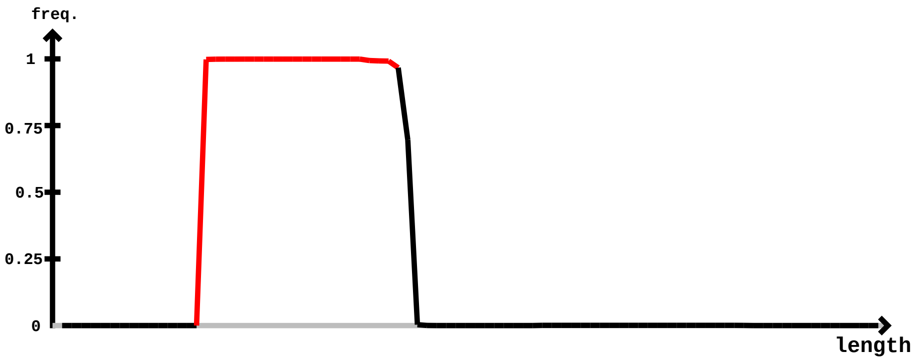


```
                oar-miR-26b
5'- ugcccgggacccaguucaaguaauucaggauagguugugugcuguccagccuguucuccauuacuuggcucgggggccggugccc   -3'    exp
    .((((((..((((((.((((((((..(((((((((((.(......))))))))))))..)))))))))))).)))..)))).))..   reads      mm         sample
    ..............uucaaguaauucaggUu......................................................   1          1          seq
    ..............uucaUguaauucaggau......................................................   4          1          seq
    ..............uucaaguaUuucaggau......................................................   2          1          seq
    ..............uucaaguaauucaggau......................................................   557        0          seq
    ..............uucaaguaauucaggaC......................................................   59         1          seq
    ..............uucaGguaauucaggau......................................................   3          1          seq
    ..............uucaagCaauucaggau......................................................   1          1          seq
    ..............uCcaaguaauucaggau......................................................   2          1          seq
    ..............uucaaguaauucagUau......................................................   5          1          seq
    ..............uucaaguaauucaggCu......................................................   1          1          seq
    ..............uucaaguaauucaggaG......................................................   5          1          seq
    ..............uucCaguaauucaggau......................................................   23         1          seq
    ..............uucaaguaauucaggaA......................................................   17         1          seq
    ..............uucaaguGauucaggau......................................................   3          1          seq
    ..............uucaaguaauucagAau......................................................   1          1          seq
    ..............uucaaguaauucaggaua.....................................................   165        0          seq
    ..............uucCaguaauucaggaua.....................................................   7          1          seq
    ..............uucaaguaauucaggaAa.....................................................   3          1          seq
    ..............uucaaguaauucagAaua.....................................................   1          1          seq
    ..............uucaaguaauucaggauag....................................................   96         0          seq
    ..............uucaaguaauucaggauagg...................................................   3161       0          seq
    ..............uucaaguaauucaggauaggu..................................................   34612      0          seq
    ..............uucaaguaauucaggauagguu.................................................   88381      0          seq
    ..............uucaaguaauucaggauagguug................................................   379        0          seq
    ..............uucaaguaauucaggauagguugu...............................................   51         0          seq
    ...............ucaaguaauucaggaua.....................................................   1          0          seq
    ...............ucaaguaauucaggauagg...................................................   9          0          seq
    ...............ucaaguaauucaggauaggu..................................................   32         0          seq
    ...............ucaaguaauucaggauagguu.................................................   85         0          seq
    ...............ucaaguaauucaggauagguug................................................   5          0          seq
    ...............ucaaguaauucaggauagguugu...............................................   1          0          seq
    ................caaguaauucaggauagg...................................................   1          0          seq
    ................caaguaauucaggauaggu..................................................   4          0          seq
    ................caaguaauucaggauagguu.................................................   13         0          seq
    .................aaguaauucaggauaggu..................................................   1          0          seq
```

oar-miR-26b

```
ugcccgggacccaguucaaguaauucaggauagguugugugcuguccagccuguucuccauuacuuggcucgggggccggugccc
.................aaguaauucaggauagguu.................................................    9     0     seq
..................aguaauucaggauaggu..................................................    1     0     seq
...................guaauucaggauagguu.................................................    1     0     seq
....................................gugugcuguccagccuguucuccauuacuuggcu...............    1     0     seq
................................................gccuguucuccauuacuuggcu...............    1     0     seq
.................................................ccuguucuccauuacuugg.................    1     0     seq
.................................................ccuguucuccauuacuuggc................    3     0     seq
.................................................ccuguucuccauuacuuggcu...............    42    0     seq
.................................................ccuguucuccauuacuuggcuc..............    56    0     seq
.................................................ccuguucuccauuacuuggcucg.............    3     0     seq
```
